# Topographic, Thermal and Chemical Characterization of Oxidized Cu and Cu-Ag Thin Films

**DOI:** 10.3390/ma18194562

**Published:** 2025-09-30

**Authors:** Maria C. Carrupt, Ana M. Ferraria, Ana P. Serro, Ana P. Piedade

**Affiliations:** 1 CEMMPRE, Department of Mechanical Engineering, University of Coimbra, 3030-788 Coimbra, Portugal; m.cristina_borges@hotmail.com; 2 iBB, Interdisciplinary Complex, Instituto Superior Técnico, University of Lisbon, 1049-001 Lisbon, Portugal; ana.ferraria@tecnico.ulisboa.pt; 3 CQE—Instituto Superior Técnico, Department of Chemical Engineering, University of Lisbon, 1349-017 Lisbon, Portugal; anapaula.serro@tecnico.ulisboa.pt

**Keywords:** Cu-Ag thin films, natural and thermal oxidation, thermal conductivity, thermal diffusivity, thermal capacity

## Abstract

This study investigated the effects of silver doping, natural ageing, and thermal-induced oxidation on the surface chemistry, morphology, and thermal performance of copper thin films. Ag is used as a doping element in Cu because, in bulk materials it usually refines microstructures, leading to increased hardness and mechanical strength through mechanisms such as solid solution strengthening and twinning. In this work was also used due to its oxidation resistance. Thin films of pure and silver-doped copper (Cu_2Ag and Cu_4Ag) were deposited by RF magnetron sputtering and characterized as-deposited, naturally aged, at room temperature and humidity for one year, and thermally treated at 200 °C, in air. The characterization included X-ray photoelectron spectroscopy (XPS), Atomic Force microscopy (AFM), and thermal analysis, specifically thermal conductivity (λ), thermal diffusivity (α), and thermal capacity (ρ.Cp). Surface XPS analysis revealed changes in copper and silver oxidation states after natural aging and annealing. AFM revelead that the incorporation of silver and heat treatment altered the surface roughness and morphology. Thermal analysis found that for lower silver concentrations, the thermal conductivity increased, but aging and annealing had varying effects depending on the silver content. The Cu_4Ag film showed the best thermal stability after natural ageing. Overall, the results suggest that carefully controlled silver doping can enhance the thermal stability of copper thin films for applications where aging is a concern, such as microelectronics.

## 1. Introduction

Copper and silver are widely used in bulk or as coatings [1,2,3] in various applications, due to their optical, thermal, and electrical properties. In recent years, their potential has made them promising candidates for efficient, flexible, and sustainable energy devices, being explored for green energy technologies, particularly in photovoltaics and electro catalysis. Cu and Ag thin films, both individually and as alloys or dopants, are widely used to enhance solar cell performance [4]. Ag-doped Cu-based absorbers (e.g., Copper Zinc Tin Sulfide (CZTS) and Copper Indium Gallium Selenide (CIGS)) show improved open-circuit voltage, reduced defect density, and higher power conversion efficiencies, with some devices exceeding 10% efficiency [5,6,7]. Ultra-thin Ag(Cu) films serve as transparent electrodes, enabling flexible polymer solar cells with efficiencies up to 7.5% and excellent mechanical stability [8]. Cu/Ag–Sb–I and Ag-doped Cu_2_O thin films also demonstrate potential for use indoor and in silicon-based solar cells, respectively, although further efficiency improvements are needed [9,10]. Ultra-thin Cu doped Ag transparent conductive films with excellent thermal, chemical, and mechanical stability enhance the flexibility of polymer solar cells [8].

Recent research has explored the use of Cu and Ag in thin films for interconnects, transparent electrodes, flexible electronics, and photovoltaics, with a focus on optimizing performance and durability. According to Strehle [11], electroplating Cu(Ag) alloys can produce homogeneous thin films with low electrical resistivity for interconnect applications. Nanostructured micromesh Cu-Ag/indium tin oxide thin-film electrodes (TTFEs) provide highly flexible, transparent, and high-performance thin-film heaters for flexible electronics, smart windows, and wearable devices [12].

Copper and silver have been applied to improve thermal conductivity in various materials and devices, including composite structures [13]. Their high intrinsic thermal conductivities, combined with innovative fabrication and layering techniques, enable efficient heat dissipation and multifunctional performance. Silver, in particular, has been applied as a thermal stabilizer by inhibiting grain growth, reducing defect formation, and suppressing atomic diffusion at elevated temperatures [14].

Research indicates that Ag not only improves thermal stability but also influences phase transitions and grain growth in various thin film systems. Kin et al. [15] synthesized Ag-Cu double layer on the biodegradable polymer poly(lactic acid) (PLA). The composite PLA/Ag/Cu exhibited excellent thermal conductivity with only 2.4 vol%-Ag and 6.3 vol%-Cu, due to the homogeneous interconnected Cu and Ag structures. They concluded that the homogeneous Ag layer affects both the structure uniformity and oxidation resistance of Cu. In their research, Niti et al. [16] investigated the effect of Ag doping on the phase formation of iron mononitride (FeN) thin films. Their results show that Ag doping significantly enhances the properties of FeN thin films, improving their thermal stability and reducing structural defects. Ag doping also reduced Fe self-diffusion, thereby improving the thermal stability.

Herein, we investigated how doping copper thin films with 20 at.% and 40 at.% of silver (Cu_2Ag and Cu_4Ag, respectively) affects their surface, morphology, and thermal properties. Thin films of pure copper and silver-doped copper were deposited by RF magnetron sputtering on silicon and steel substrates. The films were characterized in their as-deposited condition, after annealing (200 °C for 30 min in air, open furnace), and after natural aging (1 year) using X-ray photoelectron spectroscopy (XPS), atomic force microscopy (AFM), and thermal constant analysis techniques (HotDisk^®^).

Overall results show that the surface chemical composition and roughness were affected by annealing. The effects of silver doping on thermal conductivity varied between the as-deposited and treated thin films. The results suggest that carefully controlled silver doping can enhance the thermal stability of the copper thin films, particularly for applications where long-term ageing is a concern. However, the effects depend strongly on the silver concentration and thermal history. This study offers insights into optimizing the composition and processing of Cu-Ag thin films to enhance their reliability in applications where thermal conductivity and thermal stability are necessary.

## 2. Materials and Methods

### 2.1. Depositions and Oxidation of Thin Films

**Figure 1:**
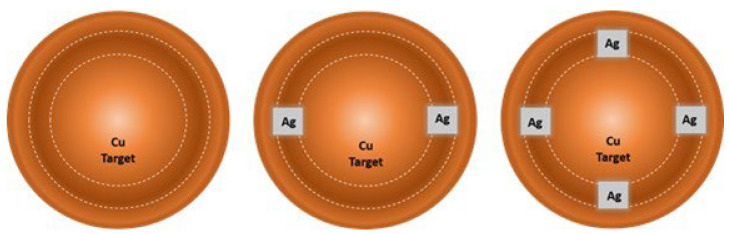
Schematic representation of silver coupons placed over the highest erosion zone of the Cu target.

Thin films of copper and silver-doped copper were deposited on silicon and AISI P20 steel as described in a previous work [17], using a pure copper target doped with 2 or 4 silver coupons of 10 × 10 mm^2^, placed on the area of high erosion (Figure 1). Coated silicon was used for characterization by X-ray photoelectron spectroscopy (XPS) and atomic force microscopy (AFM). The thermal conductivity, thermal diffusivity, and thermal capacity of films were measured on coated steel. Before the deposition, all substrates were ultrasonically cleaned in acetone and alcohol for 10 min in each.

Three types of thin films were deposited: (i) pure copper (Cu); (ii) Ag-doped Cu, co-deposited with two pieces of silver (Cu_2Ag—20 at.%Ag); and (iii) Ag-doped Cu, co-deposited with four pieces of silver (Cu_4Ag—40 at.%Ag).

According to previously published work [17], the as-deposited doped coatings have a chemical composition of 20 at.% of Ag and 40 at.% of Ag, and were designated as Cu_2Ag and Cu_4Ag. The previous characterization revelead that all thin films have a nanometric thickness (Table 1), nanometric grain size and strong crystallographic orientation along the (111) diffraction plane of copper. After deposition, the coated samples were divided into three groups: in the first, they were kept as-deposited (AD); in the second group, they were annealed for 30 min at 200 °C (HT30); the third group was composed of samples as-deposited, left exposed to natural temperature and humidity conditions for one year (AD (1Y)) and then annealed according to previous conditions (HT30 (1Y)). The results of Table 1 also allow us to determine the average value of the oxide layer after the thermal annealing, being 340 nm for Cu, 260 nm for Cu_2Ag, and 80 nm for Cu_4Ag. These results were determined from the SEM micrographs of the thin films’ cross-sections as published in previous work [17].

The results show that the thickness of Cu and Cu_2Ag more than doubled after heat treatment. As discussed in our previous published work [17], there are two possible causes for this: grain growth and/or oxidation. Considering that the grain growth occurs for Cu-Ag films from a temperature of 180 °C, and the growth is controlled by the diffusion of silver atoms to the grain boundary, pinning it and hindering grain growth during the heating, it is expected that the grain growth in Cu and Cu_2Ag occurs more freely than in Cu_4Ag. Since silver oxide is thermodynamically less likely to occur than copper oxides, increasing the Ag content leads to a thinner oxidation layer forming during heat treatment. Consequently, the increase in thickness due to the treatment is linear with the decrease in Ag content.

### 2.2. Characterization Techniques

#### 2.2.1. X-Ray Photoelectron Spectroscopy (XPS)

The XPS analysis was performed on samples coated on silicon to evaluate the surface conditions in terms of the presence of oxides and valence states of Cu and Ag. This analysis was carried out using a non-monochromatic spectrometer, XSAM800 of Kratos (Dias de Sousa, Portugal), with an aluminium anode (hν = 1486.6 eV), take-off angle = 0°, 10 mA, 12 kV. The correction of charge deviation was done using the binding energy of aliphatic carbon (285 eV) as a reference. The software XPSPEAK 4.1 was used to make the deconvolution and identify the peaks. The following sensitivity factors were applied to quantitative analysis: 0.278—C 1s; 0.78—O 1s; 5.321—Cu 2p_3/2_; 5.987—Ag 3d.

#### 2.2.2. Atomic Force Microscopy (AFM)

The AFM analysis was performed for a morphological/topographical analysis of the surfaces. The characterization was made using a Bruker Innova System in tapping mode with a Si probe having a tip radius of 8 nm, a spring resonant frequency of 300 kHz, and a force of 40 N/m. The measurements were conducted in air at a controlled room temperature and humidity of 21 °C and 35%, respectively. Seven locations of 2 × 2 µm^2^ area per sample were chosen for collecting images, which were then treated with Gwyddion 2.64.

#### 2.2.3. Thermal Constants Analysis (Hot Disk^®^)

A Thermal Constants Analyser TPS 2500 S (Hot Disk^®^), disk type Kapton 5501 F1, was employed to measure the thermal conductivity (λ), thermal diffusivity (α), and thermal capacity (ρ.Cp) of the films at a constant room temperature and humidity of 21 °C and 35%, respectively. The transient plane source method involved placing a 6.4 mm diameter sensor, Kapton 5501 F, 6.9 Ω, between identical coated steel samples, and applying 1 W of power for 20 s.

**Figure 2:**
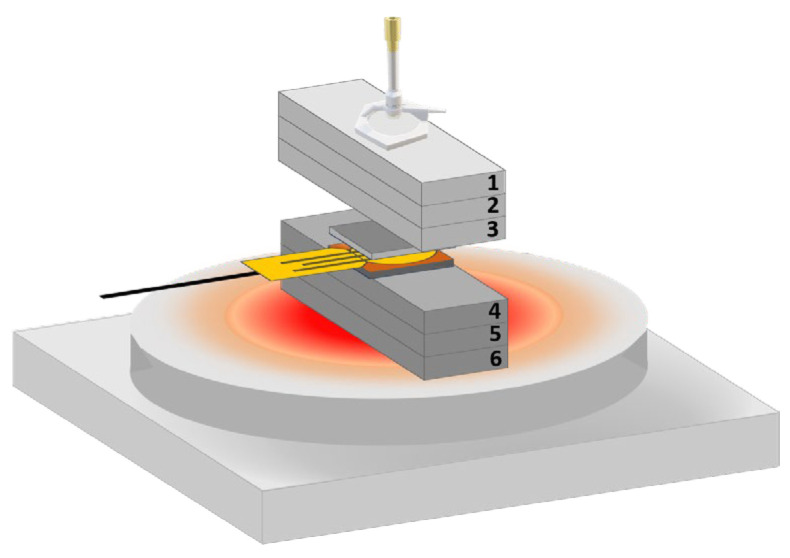
Schematic representation of the sample assembly for the thermal analysis on Hot Disk^®^.

Due to the dimensions of the coated samples, 25 × 25 × 2 mm^3^, it was necessary to insert them between pieces of the same steel used for the substrate. Six individual parts, each measuring 75 × 25 × 5 mm^3^, were stacked in groups of three to increase the overall thickness. These pieces were numbered to keep the same configuration in all measurements (Figure 2). Three measurements were taken for each sample, and the average value was calculated. This configuration enabled a comparative evaluation of the thermal characteristics of films, since their thickness is submicrometric (Table 1).

## 3. Results

The analyses of SEM (Scanning Electron Microscopy), XRD (X-ray Diffraction), EDS (Energy-Dispersive X-ray Spectroscopy), and micro-Raman mentioned in this section, were previously obtained and discussed in detail in a earlier publication of some of the authors [17].

### 3.1. XPS Analysis

**Figure 3:**
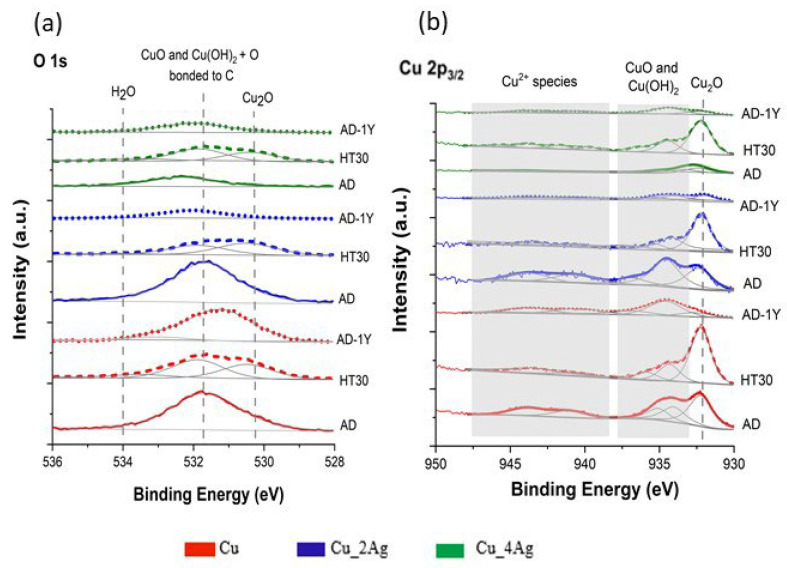
Deconvolution of (**a**) O 1s and (**b**) Cu 2p3/2 XPS spectra of pure Cu and Ag-doped Cu thin films (Cu_2Ag and Cu_4Ag) films, at different conditions: as-deposited (AD), after heat treatment (HT30), and after one year of exposed to natural temperature and humidity (AD-1Y).

**Figure 4:**
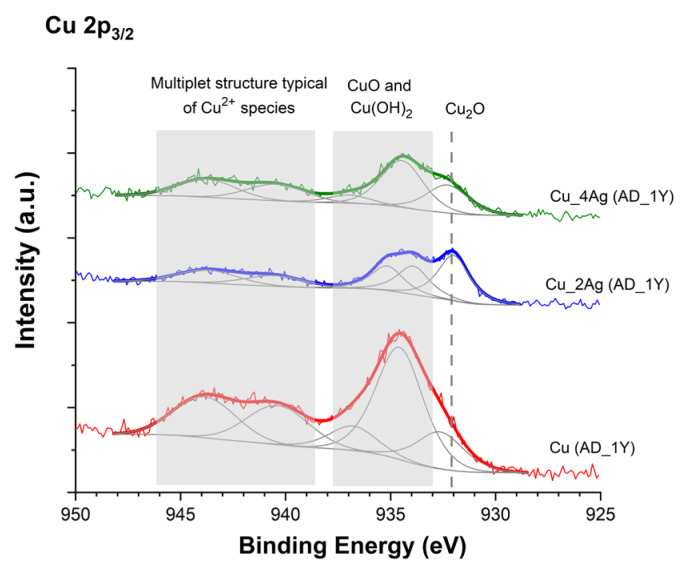
Detail of fitting of Cu 2p3/2 XPS spectra of Cu and Ag-Cu thin films (Cu_2Ag and Cu_4Ag) after one year of exposure to natural temperature and humidity conditions (AD-1Y).

XPS analyzed the valence states of the chemical elements that constitute the thin films, and the results are presented in Figure 3 (O 1s spectra and Cu 2p_3/2_ spectra) and Figure 4 (Ag 3d spectra), for all conditions of the studied thin films. The oxygen is present in all the studied surfaces forming CuO and Cu_2_O (Figure 3a) as expected, since the formation of these oxides is thermodynamically more favourable than silver oxides. This fact can be confirmed by comparing the standard enthalpy formation of CuO (−155 kJ/mol) and Cu_2_O (−168.6 kJ/mol) in comparison with Ag_2_O (−31.0 kJ/mol) [18].

According to the literature [19,20], the peak at 531.7 eV is responsible for the Cu contamination from air humidity (CuO + Cu(OH)_2_), and its oxidation as Cu_2_O (530.3 eV). The oxidation of Cu to Cu_2_O begins immediately upon the sample’s exposure to ambient air [19]. This peak (531.7 eV) is more intense for as-deposited (AD) Cu pure film, as-deposited after one year of natural aging (AD-1Y) conditions, and for as-deposited Ag-doped Cu film Cu_2Ag (AD). It can be inferred that this behavior of the Cu_2Ag surface is due to its copper content. According to the previous EDS analysis of these thin films [17], the atomic percentage of Cu on Cu_2Ag and Cu_4Ag is 82.5% and 61.2%, respectively. Due to the smaller copper content, the Cu_4Ag film (AD) shows lower contamination by CuO + Cu(OH)_2_ and H_2_O, in the region of 532–534 eV, without the formation of Cu_2_O.

After one year of exposure to natural aging conditions, there are almost no changes in that region of the spectra for Cu (AD-1Y). The same is observed in the surface of the as-deposited Cu_4Ag after 1 year (AD-1Y) compared with the as-deposited Cu_4Ag (AD) thin film. On the other hand, the Cu_2Ag film shows a substantial decrease in the intensity of the CuO + Cu(OH)_2_ peak (531.7 eV) without an increase in the Cu_2_O band (530.3 eV). Although these results contrast with those of Timalsina et al. [21], they agree with those of Platzman et al. [19], which affirmed that the oxidation of Cu to Cu_2_O is very fast on the first days. After approximately two months, the oxidation layer becomes stable (passivation), similar to the first stages, resulting in Cu(OH)_2_ formation.

In the annealed samples at 200 °C (HT30), the O 1s spectra show an increase in the intensity of the Cu_2_O peak (530.3 eV), and a decrease in CuO + Cu(OH)_2_ peak (531.7 eV) in all surfaces after annealing, indicating that exposure to temperature and air promoted an increase in the absorption of oxygen atoms on the surface of the films by diffusion, favoring the oxidation reaction with a simultaneous desorption of OH^−^. This effect is more evident in the Cu 2p_3/2_ spectra. A similar effect was observed in AgCu films by Pal and Mohan [20], which was explained by the thermodynamic affinity of Cu for oxygen as referred earlier.

The Cu 2p_3/2_ spectra (Figure 3b) show a peak at a binding energy of 932.3 eV for Cu_2_O, a peak at a binding energy of 934.6 eV with a shoulder at 937.3 eV and 933.4 eV corresponding to CuO + Cu(OH)_2_, and peaks between 938 eV and 947 eV corresponding to Cu^2+^, for all films. Comparing the fitting spectra of films as-deposited (AD) with those of annealed thin films (HT30), an intensity increase in the peak of Cu_2_O against a decrease in the peak corresponding to CuO + Cu(OH)_2_ can be observed. A decrease in the intensity of the Cu^2+^ spectrum was noted. As mentioned earlier, the annealing temperature (200 °C) enhances the absorption of oxygen on the surface and facilitates the transport of copper ions from the metal to the oxide-oxygen interface through diffusion [19].

Figure 4 shows the detailed fitting spectrum of the films as deposited and after natural aging, where the region of the Cu^2+^ species is clearly visible. Comparing the fitting spectra of films as-deposited (AD) with spectra of films as-deposited after natural aging (AD-1Y), we see an intensity decrease for both Cu_2_O and CuO + Cu(OH)_2_ peaks for Cu and Cu_2Ag films. For Cu_4Ag film, a discreet increase in CuO + Cu(OH)_2_ occurs, indicating that the presence of 40 at.% of silver jeopardizes the oxidation process and, consequently, the formation of Cu_2_O with the consequent protection of the thin film from oxidation.

At room temperature and under natural humidity conditions, the oxidation of thin films exposed to natural aging for one year is driven by forces based on an induced electric field formed between the metal and adsorbed oxygen, allowing the movement of metal ions towards the oxide/air interface [21,22]. This ionic transport starts the initial oxidation and attenuates as the thickness of the oxide layer increases [19]. The concentration of oxygen adsorbed depends on the surface properties/characteristics of the thin films, such as topography, roughness, lattice defects, and chemical composition [19,21,23]. For the Cu-Ag surfaces, it is evident that the lower thermodynamic affinity of Ag toward oxygen disrupts the normal formation of the most stable copper oxide.

**Figure 5:**
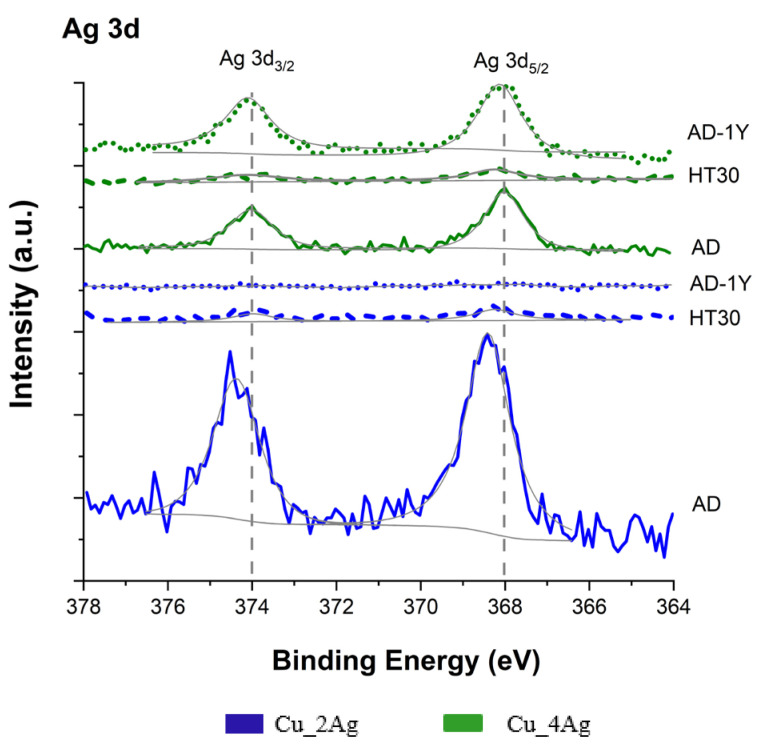
Fitting of Ag 3d3/2 and Ag 3d5/2 XPS spectra of Cu film and silver-doped copper (Cu_2Ag and Cu_4Ag) films, at different conditions: as-deposited (AD), after heat treatment (HT30), and after one year of exposed to natural temperature and humidity (AD-1Y).

The Ag 3d spectrum (Figure 5) shows twin peaks at binding energies of 368 eV and 374 eV for Cu_4Ag (AD) and Cu_4Ag (AD-1Y), respectively, corresponding to binding energies of Ag 3d5/2 and Ag 3d3/2, respectively. The fitting spectrum for Cu_2Ag (AD) has the same peaks that correspond to Ag 3d5/2 and Ag 3d3/2, but they are slightly displaced to 368.4 eV and 374.5 eV. These values are in agreement with elemental metallic Ag and suggest that, despite the presence of Ag on the surface, the level of Ag oxidation is not significant in these films [20]. Natural aging does not affect the oxidation of silver for Cu_4Ag surface. On the other hand, the peaks of Ag 3d5/2 and Ag 3d3/2 are not detected in Cu_2Ag (AD-1Y), indicating the absence of silver on the surface, likely due to the formation of a copper oxide layer. The oxidation of Ag becomes evident when it is annealed above 200 °C [17], which was not the case.

The spectrum of both Cu_2Ag and Cu_4Ag thermally annealed does not show peaks at binding energies for Ag 3d5/2 and Ag 3d3/2. Also, the Auger signal for Ag was not detected (or it is weak), which suggests that the silver is below the depth reachead by the characterization under these XPS conditions.

Unlike what occurs with naturally oxidized (aged) Cu_2Ag (AD-1Y), the heat treatment promoted mass diffusion of Ag into the film. This process is known as agglomeration and has been observed in many Ag films for substrates at high temperatures [24]. The agglomeration of Ag is a thermally activated process in which the total energy of the film is minimized by continuous dewetting and gradual exposure of the substrate. This process begins with grain boundary grooving and is followed by the formation of voids, hillocks, and islands, which reduces the total energy of the system [24,25]. In a previous characterization, a decrease in surface energy for Cu_2Ag and Cu_4Ag after annealing was observed [17].

The dewetting and resulting agglomeration are attributed to the compressive stress generated by the phase change that occurs during heating, such as the transformation from initially amorphous phases to a polycrystalline phase, which leads to an increase in volume [24]. This behavior is in agreement with previous XRD results [17], where the Ag diffraction peaks are not visible in the as-deposited (AD) and as-deposited after natural aging (AD-1Y) samples, indicating that silver may present as an amorphous structure under these conditions. Nonetheless, after heat treatment, the diffractogram shows the diffraction peaks of Ag and a change in the intensity of the (111) diffraction crystallographic plane of Cu.

### 3.2. AFM Characterization

**Figure 6:**
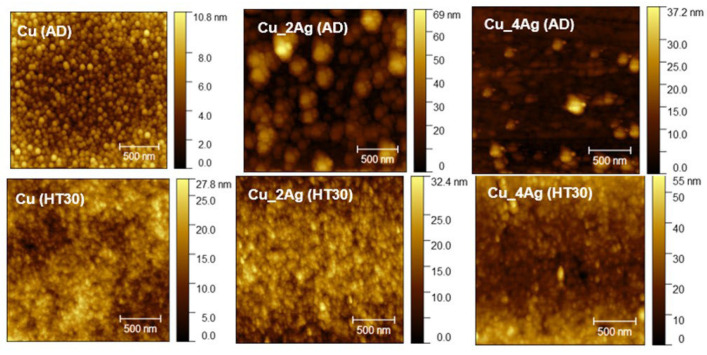
AFM topographic images of the surfaces of Cu, Cu_2Ag, and Cu_4Ag thin films, as-deposited (AD) and after annealing at 200 °C for 30 min (HT30).

**Figure 7:**
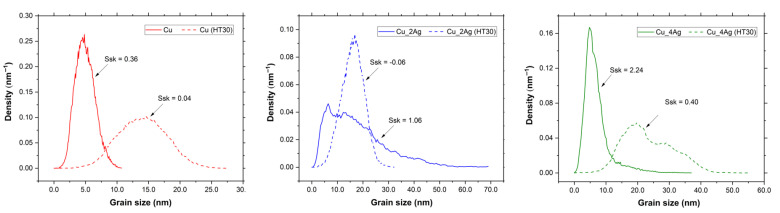
A representation of the density of grains distributed according to their size, before and after annealing (HT30), for Cu, Cu_2Ag, and Cu_4Ag thin films, and the Skewness (Ssk) parameter.

**Figure 8:**
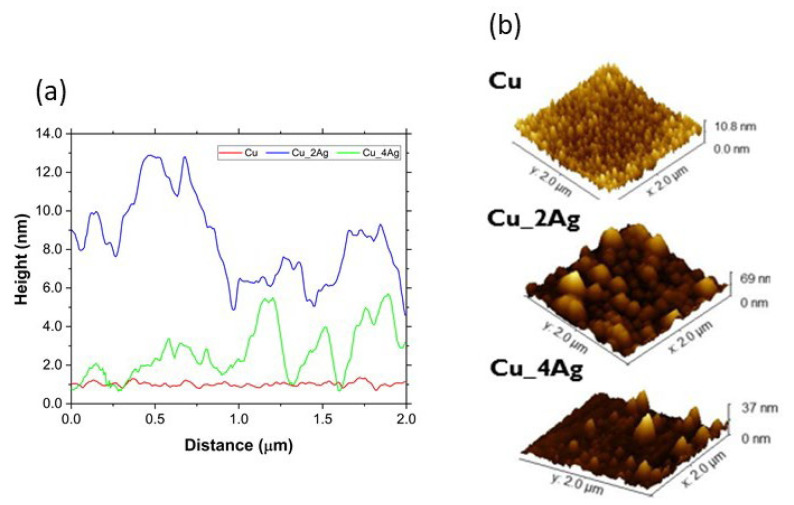
Surface roughness (Sa) of as-deposited (AD) thin films: (**a**) a graphic comparing the 2D-surface roughness (Ra); (**b**) a 3D view of the surface from AFM analysis, by Gwyddion.

**Figure 9:**
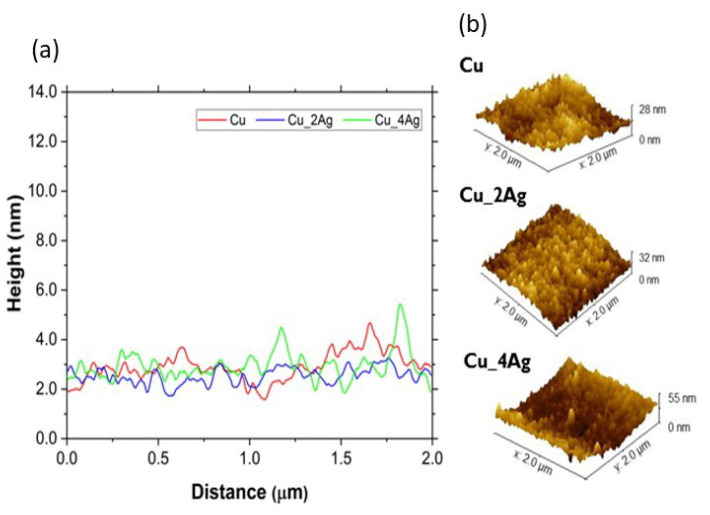
Representative surface roughness surface (Sa) of films after annealing at 200 °C for 30 min (HT30). (**a**) a graphic comparing the surface roughness (Sa) along the distance; (**b**) a 3D view of the surface from AFM analysis by Gwyddion.

The surface morphology of the coatings, as-deposited and annealed, deposited on the silicon substrate, was analyzed by AFM to determine the roughness and to infer the formation of distinct compounds or precipitates, due to the addition of silver and the aging process. The images are shown in Figure 6. Figure 7 illustrates a representation of grain distribution, categorized by size, derived from Gwyddion analysis. Figure 8 and Figure 9 illustrate the effect of annealing on the surface morphology of the films.

In the as-deposited condition (AD), we observe that the surface of the copper film is more homogeneous and smoother than the doped surfaces (Figure 6), with the highest density of peaks with a height of 5 nm (varying 0–10.8 nm).

With the addition of silver, the surface of Cu_2Ag becomes irregular due to the changes in grain distribution (Figure 7). Although Figure 6 shows that the sizes (height) vary from 0 nm to 69 nm, the highest density of grain sizes is distributed between 0 and 30 nm sizes (with an average height of around 15 nm), with a small part above this value, as shown in Figure 7. The Cu_4Ag films exhibit a normal grain size distribution, similar to that of the Cu film, with an average size of approximately 5 nm (Figure 7). A small percentage of grain has sizes between 10 and 39 nm.

The Cu film has a normal grain size distribution before and after annealing, with a skewness (Ssk) close to zero for both, indicating a balance in the number of peaks and valleys (Figure 7). On silver-doped copper films, annealing at 200 °C results in a change in surface morphology to a regular size distribution, especially for Cu_2Ag. The Skewness (Ssk) values of these films are higher than zero (Ssk > 0) before heat treatment, indicating the dominance of peaks (Figure 8). After annealing, the values of Ssk remain positive. However, they decrease to values very close to zero (Figure 7), indicating a balance between peaks and valleys and making the surface roughness of silver-doped copper films smoother (Figure 9).

The comparison between Figure 8 and Figure 9 confirms that annealing, despite promoting grain growth, has made the surfaces of the silver-doped copper films smoother, with the greatest impact on the Cu_2Ag film, as discussed previously and displayed in Figure 7.

The values of average roughness surface (Sa) and Root Mean Square (RMS) roughness are shown in Table 2.

According to the literature [26,27,28,29,30,31,32,33,34,35], the mechanisms by which annealing affects the surface roughness in Cu-Ag thin films can be grain growth and recrystallization, texture evolution, crystallographic state, mass transport and surface diffusion, agglomeration, phase segregation, surface energy minimization, and chemical composition.

Since the deposition occurred at a low temperature, due to the water-cooled substrate, the thermal energy of the arriving adatoms during the deposition was not enough to structurally organize silver, as it stayed in an amorphous or quasi-amorphous state [4,16,17]. With the annealing process, the organization of silver in crystalline phases occurs, as it was observed in the previous XRD analysis of these films [17]. The literature states that this phenomenon promotes changes in morphology and phase distribution, resulting in smoother surfaces depending on the Cu-Ag compositions [26]. The annealing also promotes grain growth and surface diffusion of silver along grain boundaries, making them denser, which results in smoother surfaces due to the reduction in defects and promotion of more uniform microstructural features. Additionally, the presence of copper oxides results in a reduction in surface energy [27,28,29].

Since the XPS analysis did not identify the presence of silver on the surface of the annealed films (Figure 5), in agreement to previous characterization [17], expanding the possibility of increasing the presence of the copper oxides, which are responsible for the decrease in the surface energy of all films after annealing (Table 3). This corroborates the presence of oxides, copper ones, a ceramic material that presents a lower surface energy than their metallic surfaces counterparts [36].

In terms of roughness, the Cu_2Ag was affected by annealing differently from the Cu pure and Cu_4Ag films. The Cu_2Ag roughness decreases while the Cu and Cu_4Ag roughness increase after annealing. The roughness can be affected by annealing because it promotes grain growth. The grain growth reduces the grain boundary area, implying a decrease in boundary defect density, which results in a decrease in surface roughness [37]. Nonetheless, it seems that with a concentration of 20 at.% of silver, the thin films have an opposite behavior, perhaps due to the ongoing accommodation of atoms with a higher atomic radius (Ag = 0.144 nm and Cu = 0.128 nm [18]), leading to a distortion in the crystalline network of Cu, which may contribute to the roughness increase. These results indicate that an optimal concentration of the doping element must be achieved to attain smoother surfaces, resulting in lower defect density in the grain boundaries.

In fact, previous research indicates that the presence of Ag, at specific concentrations, may have promoted the anchoring of grain boundaries during the annealing process [4]. According to Hung and Hsieh [26], the film Cu40Ag60 has a smaller grain size than Cu90Ag10 since the presence of the Cu phase can enhance the growth of Ag grains. Thus, films with a higher percentage of copper tend to have larger grain sizes. As mentioned earlier, the atomic percentages of copper on Cu_2Ag and Cu_4Ag are 82.5 and 61.2%, respectively.

### 3.3. Thermal Constants Analysis (Hot Disk^®^)

The thermal properties of films were analyzed by thermal constant analysis using a hot disk probe (Hot Disk*^®^*). The values of thermal conductivity (*λ*), thermal diffusivity (α), and volumetric thermal capacity (ρ.Cp) were obtained for as-deposited (AD), annealed at 200 °C for 30 min (HT30), and for natural aging conditions after one year (AD-1Y), for all films.

**Figure 10:**
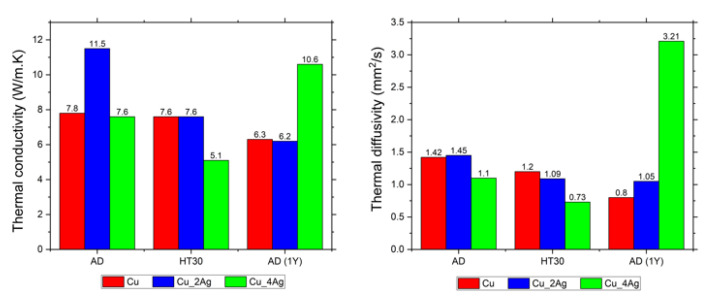
Graphics comparing thermal properties of films as-deposited (AD), after annealing at 200 °C for 30 min (HT30), and as-deposited after one year of natural aging (AD-1Y). (**Left**): Thermal conductivity (λ); (**Right**): Thermal diffusivity (α).

The values of thermal conductivity and thermal diffusivity are plotted in graphics in Figure 10. The thermal diffusivity is a thermophysical property of a material that establishes a relationship between thermal conductivity and volumetric thermal capacity (the ability to maintain heat). This relationship is described by Equation (1). In other words, it describes how quickly heat transfers through the material. Values of diffusivity α > 1 indicate that the response of the material to a temperature change is fast. For values of diffusivity α < 1, the opposite is true.

(1) $$\alpha =\frac{\lambda }{\rho Cp}$$

Table 4 shows the values of thermal conductivity (λ), thermal diffusivity (α), and volumetric thermal capacity (ρ.Cp).

The thermal conductivity of the Cu thin film was affected by aging, as expected, according to previous results. The XPS results show that, in the as-deposited condition, CuO is present on the surface, and after annealing, Cu_2_O formation occurs. Due to this, the volumetric thermal capacity increases, resulting in a slight decrease in thermal conductivity and diffusivity, as expected, because the formation of copper oxides creates additional thermal barriers to heat transport [38]. The effect of natural aging (AD-1Y) was more evident. The volumetric thermal capacity increases, and the thermal diffusivity decreases to values below 1, indicating a slow response to temperature changes.

In Ag-doped Cu thin films, the Ag content was responsible for distinct behaviors. In the Cu_2Ag (AD) film, a significant portion of the silver content is concentrated on its surface, as shown in the XPS spectra (Figure 5), resulting in the highest thermal conductivity compared to the other films. However, the value of thermal diffusivity is close to that of the copper film, likely due to the high percentage of copper (≈80 at.%). After annealing, Cu_2Ag (HT30) does not show silver on its surface (Figure 5), and the thermal conductivity of Cu_2Ag (HT30) is the same as that of the Cu (HT30) film. However, Cu_2Ag has a higher volumetric thermal capacity than copper due to the formation of Ag_2_O [17]. Although this occurs in smaller quantities compared to CuO and Cu_2_O, because of the lower Ag content of this film, Ag_2_O exhibits lower thermal conductivity than both CuO and Cu_2_O [39].

Considering natural aging, after one year, the thermal conductivity of Cu_2Ag strongly reduces (11.5 to 6.2 W/mK) and, once again, the value is very close to the thermal conductivity of Cu film in the same condition. The silver present on the surface in Cu_2Ag (AD) diffuses into the film, as shown in XPS spectra (Figure 5), probably due to the formation of copper oxides, which are thermodynamically more favorable because of their enthalpy of formation (−168.6 kJ/mol for Cu_2_O, −155.2 KJ/mol for CuO, and −31.0 kJ/mol for Ag_2_O) [17,18]. Comparing the Cu_2Ag (AD-1Y) with Cu(AD-1Y), the doped coating has better volumetric thermal capacity and thermal diffusivity than Cu (AD-1Y), which may imply that the presence of silver contributes to thermally stabilizing the films.

The Cu_4Ag film exhibits different behavior compared to Cu_2Ag. In the as-deposited condition, this film has a thermal conductivity very close to that of Cu (AD) and Cu (HT30). The XPS spectra (Figure 5) indicate the presence of silver on the film surface at a lower concentration than in Cu_2Ag. Nevertheless, silver is in an amorphous state (as discussed in the previously published work), and this structural disorder increases phonon scattering by a broad range of vibrational modes, leading to a significant decrease in thermal conductivity due to the reduction in the free path of phonons [40]. According to Giri et al. [41], the interfacial disorder between amorphous Ag and crystalline Cu boundaries is traditionally assumed to be a thermal barrier to heat transfer, because the temperature drops across each interface.

**Figure 11:**
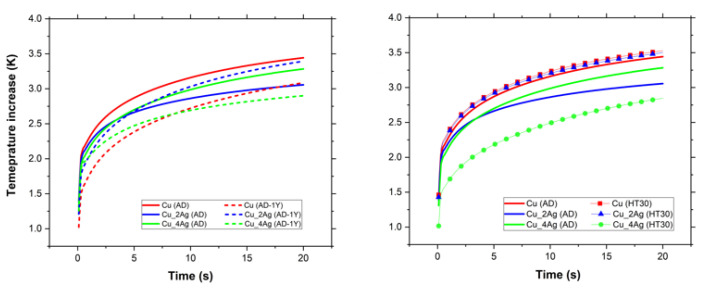
Graphics comparing the variation in temperature during a period time (thermal response). The (**left**) graphic compares the films in as-deposited (AD) conditions with as-deposited condition after one year of aging (AD-1Y). The (**right**) graph compares as-deposited (AD) with films annealed (HT30).

After annealing, no silver is observed on the surface of the film, and the Cu_4Ag (HT30) film has the lowest thermal conductivity. The formation of a higher concentration of Ag_2_O may be responsible for this. On the other hand, after one year of natural aging, Cu_4Ag (AD-1Y) has the highest thermal conductivity among all films. During this aging process, the Ag diffuses to the surface (Figure 5), promoting protection against corrosion. The thermal diffusivity also increases, giving the film the fastest thermal response. In this case, it must be considered that Cu_4Ag is composed of almost 50 at.% Ag and that this element exhibits higher thermal diffusivity than copper, in bulk, at room temperature (165 and 110 mm^2^/s, respectively [18]. The values in this work are smaller due to the nanometric dimension of the grain size, which induces a decrease in the thermal properties when compared to bulk, due to the increase in diffuse electron scattering as a consequence of an increase in total grain boundary scattering [2,42]. This behaviour was not observed in Cu_2Ag, because oxidation was provoked at the same temperature, but Ag concentration was half of the one in Cu_4Ag. Figure 11 shows comparative graphics of films in as-deposition conditions (AD) with films after annealing (HT30), and after natural aging (AD-1Y).

## 4. Conclusions

The results suggest that doping copper thin films (Cu) with silver (Ag) can enhance the thermal stability of the produced thin films, particularly in applications where aging is a significant concern.

However, when silver concentrations are high, it is crucial to closely monitor the heat treatment process, as excessive silver can negatively impact thermal conductivity due to unfavorable microstructural features. These findings are particularly relevant for advanced applications, such as microelectronics, where effective thermal management is crucial and thin metal films are commonly employed as interconnects or conductive layers.

## Figures and Tables

**Table 1:** Thickness of the thin films as-deposited (AD) and after heat treatment (HT30) [17].

	Thickness of Thin Films (nm)	
Thin Films	AD	HT30
Cu	210 ± 1	550 ± 3
Cu_2Ag	220 ± 3	480 ± 4
Cu_4Ag	240 ± 3	320 ± 4

**Table 2:** Values of average roughness surface (Sa) and Root Mean Square (RMS) roughness, quantified by Gwyddion from AFM characterization.

	As-Deposited (AD)			Annealed (HT30)		
	Cu	Cu_2Ag	Cu_4Ag	Cu	Cu_2Ag	Cu_4Ag
Sa (nm)	1.25	8.93	2.87	3.08	3.48	6.46
RMS (nm)	1.55	11.34	4.22	3.80	4.33	7.80

**Table 3:** The surface energy calculated from contact angles measured with water and formamide [17].

	Surface Energy (mJ/m^2^)		
Surfaces	Cu	Cu_2Ag	Cu_4Ag
As-Deposited (AD)	81.1	80.4	88.9
Annealed (HT30)	60.4	53.3	55.6

**Table 4:** Average and standard deviation values of thermal conductivity (λ), volumetric thermal capacity (ρ.Cp), and thermal diffusivity (α) for as-deposited (AD), annealed (HT30), and after one year of aging, the thin films.

	Thermal Conductivity (W/m.K)	Volumetric Thermal Capacity (MJ/m^3^ K)	Thermal Diffusivity (mm^2^/s)
Cu			
AD	7.8 ± 0.014	5.5 ± 0.026	1.42 ± 0.009
HT30	7.6 ± 0.009	6.4 ± 0.020	1.20 ± 0.004
AD-1Y	6.3 ± 0.082	7.8 ± 0.060	0.80 ± 0.004
Cu_2Ag			
AD	11.5 ± 0.010	7.9 ± 0.020	1.45 ± 0.003
HT30	7.6 ± 0.008	6.9 ± 0.007	1.09 ± 0.002
AD-1Y	6.2 ± 0.067	5.8 ± 0.084	1.05 ± 0.026
Cu_4Ag			
AD	7.6 ± 0.010	6.8 ± 0.023	1.10 ± 0.003
HT30	5.1 ± 0.008	6.9 ± 0.022	0.73 ± 0.003
AD-1Y	10.6 ± 0.010	3.3 ± 0.006	3.21 ± 0.008

## Data Availability

The original contributions presented in this study are included in the article. Further inquiries can be directed to the corresponding author.

## References

[1] Carvalho D., Sousa T., Morais P.V., Piedade A.P. (2016). Polymer/metal nanocomposite coating with antimicrobial activity against hospital isolated pathogen. Appl. Surf. Sci..

[2] Oliveira B.M.C., Santos R.F., Piedade A.P., Ferreira P.J., Vieira M.F. (2022). Co-W Barrier Layers for Metallization of Copper Interconnects: Thermal Performance Analysis. Nanomaterials.

[3] Piedade A.P., Vieira M.T., Martins A., Silva F. (2007). In vitro behaviour of nanocrystalline silver-sputtered thin films. Nanotechnology.

[4] Aboulfadl H., Sopiha K.V., Keller J., Larsen J.K., Scragg J.J.S., Persson C., Thuvander M., Edo M. (2021). Alkali Dispersion in (Ag, Cu)(In, Ga) Se_2_ Thin Film Solar Cells—Insight from Theory and Experiment. ACS Appl. Mater. Interfaces.

[5] Qi Y., Tian Q., Meng Y., Kou D., Zhou Z., Zhou W., Wu S. (2017). Elemental Precursor Solution Processed (Cu_1−_). ACS Appl. Mater. Interfaces.

[6] Nguyen T.H., Kawaguchi T., Chantana J., Minemoto T., Harada T., Nakanishi S., Ikeda S. (2018). Structural and Solar Cell Properties of a Ag-Containing Cu_2_ ZnSnS_4_ Thin Film Derived from Spray Pyrolysis. ACS Appl. Mater. Interfaces.

[7] Kangsabanik M., Gayen R.N. (2023). A Comprehensive Review on the Recent Strategy of Cation Substitution in CZTS (Se) Thin Films to Achieve Highly Efficient Kesterite Solar Cells. Sol. RRL.

[8] Huang J., Liu X., Lu Y., Zhou Y., Xu J., Li J., Wang H. (2018). Seed-layer-free growth of ultra-thin Ag transparent conductive fi lms imparts fl exibility to polymer solar cells. Sol. Energy Mater. Sol. Cells.

[9] Hooijer R., Weis A., Kaiser W., Biewald A., Patrick D., Arsatiants O., Helminger D., Dyakonov V., Hartschuh A., Mosconi E. (2023). Cu/Ag–Sb–I Rudorffite Thin Films for Photovoltaic Applications. Chem. Mater..

[10] Nihad A., Haneen K., Arif M., Agam B. (2021). Efficiency enhancement of nano structured Cu2O:Ag/laser etched silicon-thin films fabricated via vacuum thermal evaporation technique for solar cell application. Optik.

[11] Strehle S., Menzel S., Bartha J.W., Wetzig K. (2010). Microelectronic Engineering Electroplating of Cu (Ag) thin films for interconnect applications. Microelectron. Eng..

[12] Han S., Ju S., Jung D., Hoon S., Lee H., Yang C. (2023). Highly flexible and transparent electrodes for high-performance thin-film heaters with nanostructured micromesh Cu—Ag ultrathin films. Thin Solid Film..

[13] Pinho A.C., Morais P.V., Pereira M.F., Piedade A.P. (2025). Changes in the Antibacterial Performance of Polymer-Based Nanocomposites Induced by Additive Manufacturing Processing. Polymers.

[14] Baburin A.S., Moskalev D.O., Lotkov E.S., Sorokina O.S., Baklykov D.A., Avdeev S.S., Buzaverov K.A., Yankovskii G.M., Baryshev A.V., Rodionov I.A. (2023). Evolutionary selection growth of silver films for low-loss nanophotonic devices. Surf. Interfaces.

[15] Kim D., Lee Y.J., Ahn K.H. (2022). Interconnected network of Ag and Cu in bioplastics for ultrahigh electromagnetic interference shielding efficiency with high thermal conductivity. Compos. Commun..

[16] Niti N., Kumar Y., Seema S., Reddy V.R., Vas J.V., Gupta S., Stahn J., Gupta A., Gupta M. (2022). Stabilizing effects of Ag doping on structure and thermal stability of FeN thin films. J. Phys. Condens. Matter.

[17] Carrupt M.C., Serro A.P., Piedade A.P. (2024). Influence of the Ag Content on the Natural and Thermal Induced Oxidation of Cu Thin Films. Materials.

[18] Haynes W.M., Lide D.R., Bruno T.J. (2014). CRC Handbook of Chemistry and Physics.

[19] Platzman I., Brener R., Haick H., Tannenbaum R. (2008). Oxidation of polycrystalline copper thin films at ambient conditions. J. Phys. Chem. C.

[20] Pal A.K., Bharathi Mohan D. (2017). SERS enhancement, sensitivity and homogeneity studies on bi-metallic Ag-Cu films through tuning of broad band SPR towards red region. J. Alloys Compd..

[21] Timalsina Y.P., Washington M., Wang G.C., Lu T.M. (2016). Slow oxidation kinetics in an epitaxial copper(1 0 0) film. Appl. Surf. Sci..

[22] Ramsey J.A., Garlick G.F.J., Roberts J.K. (1949). Theory of the oxidation of metals Some interactions of gases with metals and crystalline solids. Rep. Prog. Phys..

[23] Lim J.W., Mimura K., Miyake K., Yamashita M., Isshiki M. (2003). Effect of substrate bias voltage on the purity of Cu films deposited by non-mass separated ion beam deposition. Thin Solid Film..

[24] Mohanty B.C., Malar P., Osipowicz T., Murty B.S., Varma S., Kasiviswanathan S. (2009). Characterization of silver selenide thin films grown on Cr-covered Si substrates. Surf. Interface Anal..

[25] Adams D., Alford T.L., Mayer J.W., Springer Science & Business Media (2007). Silver Metallization: Stability and Reliability.

[26] Hsieh J., Hung S. (2016). The effect of cu: Ag atomic ratio on the properties of sputtered cu-ag alloy thin films. Materials.

[27] Han H., Alford T.L. (2008). Texture and surface morphology evolution of Ag(Cu) layers on indium tin oxide thin films. J. Phys. D Appl. Phys..

[28] Han H., Zoo Y., Mayer J.W., Alford T.L. (2007). Improved surface morphology and texture of Ag films on indium tin oxide via Cu additions. J. Appl. Phys..

[29] Ţălu Ş., Matos R.S., Pinto E.P., Rezaee S., Mardani M. (2020). Stereometric and fractal analysis of sputtered Ag-Cu thin films. Surf. Interfaces.

[30] Warren A.P., Sun T., Yao B., Barmak K., Toney M.F., Coffey K.R. (2012). Evolution of nanoscale roughness in Cu/SiO_2_ and Cu/Ta interfaces. Appl. Phys. Lett..

[31] Kang S.H., Obeng Y.S., Decker M.A., Oh M., Merchant S.M., Karthikeyan S.K., Seet C.S., Oates A.S. (2001). Effect of annealing on the surface microstructural evolution and the electromigration reliability of electroplated Cu films. J. Electron. Mater..

[32] Zoo Y., Han H., Alford T.L. (2007). Copper enhanced (111) texture in silver thin films on amorphous SiO_2_. J. Appl. Phys..

[33] Filoti D.I., Bedell A.R., Harper J.M.E. (2010). Synergistic Ag (111) and Cu (111) texture evolution in phase-segregated Cu1−xAgx magnetron sputtered composite thin films. J. Vac. Sci. Technol. A Vac. Surf. Film..

[34] Purswani J.M., Gall D. (2008). Surface morphological evolution during annealing of epitaxial Cu(001) layers. J. Appl. Phys..

[35] Noorbakhsh R., Rezaee S., Nia B.A., Boochani A. (2021). Influence of deposition time on the optical and morphological properties of silver–copper thin films: Experimental and statistical studies. Opt. Quantum Electron..

[36] Swiatkowska-Warkocka Z., Shakeri M.S., Polit O., Gurgul J., Biesiadecka M., Dziedzic A., Pawlik P., Kot J. (2025). Surface Modification of CuO/Cu_2_O/Cu Composite Particles with Ag by Pulsed Laser Irradiation of Suspension and Their Antimicrobial Potentia. J. Phys. Chem. C.

[37] Das S., Alford T.L. (2013). Structural and optical properties of Ag-doped copper oxide thin films on polyethylene napthalate substrate prepared by low temperature microwave annealing. J. Appl. Phys..

[38] De Carlo I., Baudino L., Klapetek P., Serrapede M., Michieletti F., De Leo N., Pirri F., Boarino L., Lamberti A., Milano G. (2023). Electrical and Thermal Conductivities of Single CuxO Nanowires. Nanomaterials.

[39] Wang L., Sun Y., Chen Y., Wang C. (2024). Study on the Anharmonic Interaction in Negative Thermal Expansion Compounds Ag_2_O and Cu_2_O by Three-Phonon Scattering. J. Phys. Chem. C.

[40] Tanguy A. (2024). Vibrations and Heat Transfer in Glasses: The Role Played by Disorder. Comptes Rendus Phys..

[41] Giri A., King S.W., Lanford W.A., Mei A.B., Merrill D., Li L., Oviedo R., Richards J., Olson D.H., Braun J.L. (2018). Interfacial Defect Vibrations Enhance Thermal Transport in Amorphous Multilayers with Ultrahigh Thermal Boundary Conductance. Adv. Mater..

[42] Han D.-G., Yoon J.-W. (2025). Effects of the grain size and orientation of Cu on the formation and growth behavior of intermetallic compounds in Sn-Ag-Cu solder joints. J. Alloys Compd..

